# Neovascularization in Tissue Engineering

**DOI:** 10.3390/cells1041246

**Published:** 2012-12-10

**Authors:** Jennifer C.-Y. Chung, Dominique Shum-Tim

**Affiliations:** 1 Division of Cardiac Surgery, McGill University Health Center, McGill University, 687 Pine Avenue West, Suite S8.73b, Montreal H3A-1A1, Quebec, Canada; E-Mail: jennifer.chung2@mail.mcgill.ca; 2 Division of Surgical Research, McGill University Health Center, McGill University, 687 Pine Avenue West, Suite S8.73b, Montreal H3A-1A1, Quebec, Canada

**Keywords:** neovascularization, angiogenesis, therapeutic angiogenesis, endothelial progenitor cells

## Abstract

A prerequisite for successful tissue engineering is adequate vascularization that would allow tissue engineering constructs to survive and grow. Angiogenic growth factors, alone and in combination, have been used to achieve this, and gene therapy has been used as a tool to enable sustained release of these angiogenic proteins. Cell-based therapy using endothelial cells and their precursors presents an alternative approach to tackling this challenge. These studies have occurred on a background of advancements in scaffold design and assays for assessing neovascularization. Finally, several studies have already attempted to translate research in neovascularization to clinical use in the blossoming field of therapeutic angiogenesis.

## 1. Introduction

Inadequate vascularization remains one of the major challenges facing tissue engineering. Common applications of tissue engineering involve ischemic tissue (such as in myocardial infarction and peripheral vascular disease), or trauma resulting in loss of vascularization. Therefore, overcoming the challenges of neovascularization is critical in the clinical applicability of tissue engineering. Diffusion and *in vivo* capillary networks can only support tissue less than 2 mm thick, preventing practical application [1]. To allow engineered tissue or organ constructs to survive and then thrive, a comprehensive network of healthy functional blood vessels is necessary for oxygen and nutrient delivery and waste product removal. 

Angiogenesis is the development of new vessels from pre-existing blood vessels that have been converted into an angiogenic state [2]. The process of angiogenesis can occur either through intussusception or endothelial sprouting. Sprouting is a multistep process involving the dissolution of the basement membrane, the migration and proliferation of endothelial cells, the formation of a lumen and maturation of the new vessel with investment of the vessel with pericytes [3]. Intussusception involves splitting the lumen of an existing vessel into two [3].

In addition to angiogenesis, 'vasculogenesis' denotes the formation of blood vessels through the de novo differentiation of stem cells into endothelial cells such as during embryonic development [2]. This difference between angiogenesis and vasculogenesis reflects two broad categories of strategies employed by researchers, often simultaneously, to overcome the problem of inadequate vascularization; namely to improve local circulation and encourage vascular ingrowth from the recipient to the graft, or to establish new local circulation through stem cell therapy.

The present review will discuss how investigators have approached the challenge of neovascularization in tissue engineering through protein therapy and angiogenic growth factors, as well as cell therapy through endothelial cells and endothelial progenitor cells. We will discuss the various scaffolds used to deliver either proteins or cells, and how researchers evaluate the success of their neovascularization endeavors. Finally we will review how this research has translated into clinical trials, and their successes and setbacks. 

## 2. Angiogenic Growth Factors

One strategy for improving local circulation is to induce angiogenesis by manipulating pro-angiogenic growth factors. There are a number of such factors which, as the name implies, stimulate the formation of blood vessels. The most important ones include vascular endothelial growth factors (VEGF), fibroblast growth factors (FGF), platelet-derived growth factors (PDGF) and transforming growth factors (TGF). Table 1 describes important angiogenic growth factors in greater detail . VEGF represents a large family of proteins, and VEGF-A is the best studied regarding angiogenesis with its various isoforms. VEGF and FGF are potently upregulated by hypoxic conditions, and then released through perivascular extracellular matrix remodelling by matrix metalloproteinases (MMP) [4]. They increase basement membrane permeability and activate tip and stalk cells which then migrate and elongate forming a new vessel lumen [5]. These factors can be taken advantage of either by direct protein therapy or through augmenting gene expression.

Direct delivery of VEGF has been performed using intravenous and intracoronary injections in humans [6,7,8]. Unfortunately, the small amounts of VEGF that reach the area of interest using such methods fail to last more than 1 day [9]. Therefore, VEGF has been incorporated in many scaffolds of various polymers, and encapsulated in nanoparticles and microparticles [5]. For example, Wu *et al.* conjugated VEGF to a hydrogel which was then injected into a rat myocardial infarct model [10]. This produced high blood vessel density in the infarct zone and even improved ventricular function [10]. Different types of scaffolds are discussed in more detail in a later section.

**Table 1:** Important Angiogenic Growth Factors [11,12,13].

Angiogenic Growth Factor	Location	Role in angiogenesis
VEGF-A (isoforms: VEGF-A165, VEGF-A165b, VEGF-A121, VEGF-A145, VEGF-A148, VEGF-A183, VEGF-A189 and VEGF-A206)	Highly expressed in lung, kidney, heart and adrenal gland vascular smooth muscle cells, and secreted by circulating polymorphonuclear cells and platelets.	Stimulates survival and proliferation, migration and differentiation endothelial cells, increases vascular permeability.
FGF (eg. FGF-1, FGF-2)	Vascular smooth muscle cells, intestinal enterochromaffin cells, cardiac muscle cells, skeletal muscle cells	Stimulates endothelial cell survival, proliferation and migration, production of collagenase and plasminogen activator.
Platelet-derived growth factor	Platelets, fibroblasts, astrocytes, keratinocytes, epithelial cells	Upregulation of VEGF, recruitment and proliferation of pericytes and smooth muscle cells.
Angiopoietin/Tie2	Embryo: vascular endothelium, angioblasts, endocardium Adult: lung capillaries	Regulates integrity and survival of endothelial cells, regulates sprouting and branching of vessels.
Hepatocyte Growth Factor	Secreted by polymorphonuclear cells and biliary epithelial cells in the liver	Promotes survival, motility, invasion and morphogenesis of epithelial and endothelial cells
Transforming growth factor-β	Secreted by many cells including immune cells such as macrophages and plasma cells	Upregulates angiogenic factors including VEGF and FGF, and proteinases, promotes basement membrane reformation, regulates smooth muscle cell differentiation and recruitment.

Rather than directly introducing the growth factors, some investigators have opted for gene therapy. Transferring genes of pro-angiogenic factors into host cells around the area of interest theoretically leads to sustained local expression of the desired genes. Gene transfer of plasmids encoding FGF and VEGF have already been tried on patients with ischemic limb and heart disease [14,15,16,17]. Gene therapy is discussed further in the 'clinical trials' section of the article.

Despite the success of the above studies, it has become increasingly clear that successful use of angiogenic factors is incredibly complex. The exact microenvironmental dose is critical, and delivery of the most angiogenic factor that one can manage is not always the best strategy. Ozawa *et al.* illustrated this concept with a mouse model that was inoculated with myoblasts containing various degrees of VEGF expression. The group found that high expression of VEGF can actually lead to aberrant vessels and hemangiomas [18]. The balance between angiogenesis and pruning and remodelling must be respected. Moreover, it should be remembered that anti-angiogenic therapies are already currently employed to battle cancer, including glioblastoma [19], colorectal cancer [20], and hepatocellular carcinoma [21]. 

The complexity of using angiogenic factors increases beyond dosage. Their ideal use has become not only a question of which factor, but which combination of factors, and in what temporal relation are the factors to be released. Using a polymer scaffold, Richardson et al was able to release VEGF and PDGF in a sequential manner [22]. Subsequently, the sequential release of pro-angiogenic factors has also been achieved using alginate hydrogels, resulting in increased vascularization when using multiple factors as opposed to a single factor [23,24]. The recognition of complex interplay between various factors has led to the development of pro-angiogenic factor cocktails to increase the efficiency of promoting angiogenesis [25,26].

## 3. Cell Therapy

Directly culturing endothelial cells is one approach to vasculogenesis. Autologous endothelial cells in a fibrin matrix have been transplanted in a sheep ischemic heart disease model [27]. Further, cells derived from human umbilical vein endothelial cells (HUVEC) have been cultured within a collagen and fibronectin gel [28]. These cells underwent a very high rate of apoptosis, and Bcl-2 transduction was performed to overcome this. By doing so, complex microvessel systems formed and vessel maturation with smooth muscle cell recruitment was observed. The clinical applicability of this technique is hampered by the risk of tumor formation with BCL-2 overexpression. In general, mature endothelial cells have been difficult to culture in large quantities and demonstrate very limited proliferative potential [29,30].

Circulating endothelial progenitor cells (EPC) were first described in the seminal work by Asahara *et al.* in 1997 [31], who went on to demonstrate that these cells were able to augment angiogenesis as well as form blood vessels via vasculogenesis post-natally [32]. This opened the door for tissue engineers searching for a solution to the vascularization of tissue problem. EPC have been isolated, amplified and combined with Matrigel, a commercial injectable scaffold that forms a gel in the body [33]. When injected in mice, increased microvessel density was found with evidence of vessel maturation [33]. 

EPC have been isolated from both peripheral blood and the umbilical cord [34,35]. Adult bone marrow contains this useful population of progenitor cells that can mobilize once stimulated by ischemia or cytokines. Adipose tissue is an additional source with the potential to provide a minimally invasive method for isolating cells from the endothelial and EPC lineage [36].

Since the discovery of EPC, multiple populations have been described [30,37]. Late EPC, also described as outgrowth endothelial cells, differ from the original population described by Asahara *et al.* morphologically, phenotypically and in proliferative potential. Interestingly, investigators found a synergistic relationship between the two populations, generating greater capacity for neovascularization than each EPC population individually [38]. This synergism is secondary to complex paracrine signalling interactions between the two populations of EPC. However, identifying and characterizing various subsets of EPC remain major challenges [39].

In addition, the importance of perivascular cells has become well established in the formation of mature vascular networks [40,41], and smooth muscle cells can be seeded alongside EPC [33]. Thus, future use of cell therapy to overcome the obstacle of neovascularization in tissue engineering must account for the complex interplay of multiple cell types and populations. 

## 4. Hypoxia Induced Angiogenesis

Hypoxia is a well-established stimulant of angiogenesis, and the most pertinent regulator of oxygen homeostasis is the hypoxia inducible factor-1 (HIF-1) pathway [42,43]. The enzyme HIF-1 is comprised of HIF-1α and HIF-1β subunits, which are constitutively expressed, with HIF-1α being degraded under normoxia. However, under hypoxic conditions, degradation is inhibited and HIF-1 carries out its function: regulation of gene expression, specifically that of VEGF and stromal cell-derived factor (SCDF-1). SCDF-1 acts as a chemokine by binding to the receptor CXCR4 and attracts stem cells, including circulating EPC, to areas of hypoxia. Clinically, this is seen in burns, free flaps, ischemic limbs and ischemic heart disease [44,45,46,47].

Hypoxic conditions also enhances cell survival through activation of Akt, an anti-apoptotic protein, making cells such as cardiomyocytes more resistant to further ischemic insults [48]. Therefore, although typically viewed as a barrier to tissue engineering, hypoxia may have certain advantages researchers need to consider when designing a tissue construct. For example, using a process termed "hypoxic preconditioning", prior exposure of BMSC to hypoxic conditions activates Akt, leading to reduced hypoxia-related cell death, increased proliferative potential, upregulated paracrine effects, and increased secretion of angiogenic factors [48,49]. In fact, oxygen tension also plays a role in directing timing and routes of differentiation for stem cells [49]. 

Great efforts are being made to measure and control oxygen tension in bioreactors for growing tissue. It is known that 3D constructs consume more oxygen than 2D constructs, and flow perfusion alters oxygen gradients as compared to static conditions [50]. How to best manipulate oxygen tension is an ongoing area of active research in tissue engineering.

## 5. Delivery System

As previously mentioned, the delivery of angiogenic factors in a sustained manner to the area of interest and the sequential delivery of multiple angiogenic factors are major considerations for successful induction of angiogenesis via protein therapy. Meanwhile, if the cell therapy strategy is employed, one needs to ensure cell retention in the area of interest and appropriate cell release kinetics, in addition to maximizing cell survival. Systemic delivery, such as via intravascular injection, is severely limited as it would expose the host to adverse systemic effects of the angiogenic factors when usually the desired effects are local, and free protein has an abysmal elimination half life [6]. Similarly, free cell injections would fail to retain cells in the area of interest. It becomes clear that designing the appropriate cell or protein delivery vehicle is key.

Intracoronary injection was considered for delivery of angiogenic factors or angiogenic gene transfer in ischemic heart disease. The method is relatively non-invasive, can be performed at the time of coronary angioplasty, and would directly supply myocardium. However, it still exposes the patient to systemic collateral effects, fails to maintain a sustained effect, and trials have demonstrated disappointing results [7,8,51]. A possible answer to the shortfalls of systemic and intracoronary injections, is to perform gene delivery via cardiopulmonary bypass, which isolates the heart [52,53]. This would be a highly invasive procedure, but it warrants further research.

More commonly, investigators have tackled the delivery issue via scaffolds. The properties of an ideal delivery system include being composed of a biocompatible material, having the ability to retain protein and cells, and to degrade with non-toxic degradation products (be biodegradable), such that the proteins or cells are released in a sustained manner. Such scaffolds can either participate in neovascularization after implantation or *ex vivo* prior to implantation, called prevascularization. An additional approach is *in situ* vascularization, where a scaffold is remotely implanted allowing the host to form a microvasculature prior to implantation and inosculation with microvasculature at the desired location [54]. Traditionally, the scaffold is a solid or gel construct which is placed or sutured in place. Injectable scaffolds are being investigated as a less invasive alternative. Initially liquid, injectable scaffolds then form a gel once inside the body whether the phase change is secondary to body temperature [10,55,56], physiological pH [57] or UV light exposure [58]. 

The success of the scaffold requires rapid vascularization to allow survival and growth of the tissue engineering construct. It was recognized that negative space within scaffolds was needed to promote angiogenesis, and it was found that adequate pore-size (greater than 250 μm) and interconnectivity between the pores are important characteristics [59,60]. With this in mind, many materials have been used for vascularization both using natural materials such as Matrigel, as well as synthetic materials such as polymers and self-assembling nanofibres [61]. The advantage of synthetic materials is that they are highly customizable. Therefore, there is great excitement for solid free-form fabrication systems, which construct layer by layer 3D scaffolds from various materials. Examples include selective laser sintering, stereolithography and 3D printing [62]. The major consideration for synthetic materials is biocompatibility, which may require coating or additional steps in order to interact more naturally with cells [62].

Of the plethora of scaffolds available, one attractive scaffold is that of highly-customizable degradable polymer microspheres, sintered into 3D structures. They can alter protein release profiles by varying polymer ratios and alter pore sizes depending on sintering temperature [63]. Successful blood vessel growth at 3 weeks were observed when using these microspheres to seed adipose-derived stromal cells transfected with VEGF cDNA along with endothelial cells [64]. Microspheres have been used to deliver VEGF, FGF, G-CSF and mesenchymal stem cells in various animal models [65,66,67,68].

Another approach is to use the intracoronary stents that are delivered during percutaneous transcatheter angioplasty as scaffolds. Stents are routinely deployed in the treatment of myocardial infarction in areas of ischemia where neovascularization may be desirable. Paul *et al.* used this approach to line stents with nanotubes containing pro-angiogenic genes in order to improve endothelial function [69].

## 6. Evaluating Neovascularization

There have been countless methods developed to evaluate the success of neovascularization. In order to ascertain the amount and rate of neovascularization, researchers have developed both *in vivo* and *in vitro* assays. *In vivo* assays can be divided into those that use chronic transparent chambers, exterior tissue preparations or *in situ* preparations [3]. An example of a chronic transparent chamber is to implant a chamber into the ear of a rabbit, which provides a very clear preparation for microscopy [70]. However, this method is limited by being expensive and not immunologically privileged. The hamster cheek, an example of an exterior tissue preparation, is immunoprivileged but is not optically ideal due to its thickness [71]. One of the most common exterior tissue preparations is the chick chorioallantoic membrane (CAM) assay. This can be performed by creating a window in the eggshell at day 7 to day 9, inserting the graft, reattaching the eggshell and then incubating the graft [72]. Alternatively, the entire egg can be transferred to a culture dish [72]. The corneal assay, which involves introducing the test substance into a surgically created pocket in a mouse cornea, is an example of an *in situ* preparation and is one of the most common angiogenesis assays in animals [73]. 

Most models evaluating angiogenesis, including the assays above, require extrinsic vascularization, which depends on diffusion and host vascular ingrowth. Some groups have opted for an intrinsic vascularization approach, which uses an existing circulation within the construct being evaluated. The arteriovenous loop model was first described in 1979, when the surgical creation of an arteriovenous fistula produced capillary bed formation [74]. Following this, groups have embedded the arteriovenous loop in protected chambers, within which a variety of constructs can be conveniently tested *in vivo*, and a multitude of tests can be conducted once the chamber is explanted [75,76].

*In vitro* assays have the advantage of being less expensive and more amenable to high outputs. However, given the many great differences between *in vitro* and *in vivo* environments, one must be careful not to over-interpret the results of *in vitro* assays. One simple method of quantifying angiogenesis is to analyze endothelial cell proliferation through the incorporation of radioactive thymidine or BrdU during DNA synthesis [77]. However, more than proliferation, researchers are interested in whether blood vessels are formed. Cells are cultured in various 3D scaffolds such as collagen, fibrin and Matrigel. Sections of the scaffold with cells undergo immunohistochemical staining, and vessel density is then quantified by counting the number of blood vessels containing red blood cells present per high power field [22,33,78].

The list of assays used for evaluating neovascularization is beyond the scope of this review [72,77], and no gold-standard assay has emerged. It is important to recognize that as advances are being made in neovascularization, advances are also being made on how to assess the quantity and quality of the neovascularization. The quality of neovascularization is critical. When evaluating studies and trials, it is important to note that the goal is not simply to improve collateral or capillary density which may occur even in the setting of inflammation, but to develop quality vessels that have a chance to affect hard endpoints such as tissue survival.

## 7. Clinical Trials

The goals of angiogenesis in tissue engineering are analogous to those of the field of therapeutic angiogenesis. Therapeutic angiogenesis aims to direct growth of blood vessels to revascularize ischemic diseases, specifically myocardial infarction and peripheral vascular disease. Therefore, the results from clinical trials in these areas are directly relevant to the future of the clinical application of angiogenesis in tissue engineering.

The landmark trial by Schumacher et al in 1998 was the first translation of angiogenesis to the clinical setting [79]. They conducted a randomized placebo-controlled trial with the intra-myocardial injection of FGF-1 or placebo during coronary artery bypass surgery. The injection site was distal to the anastomosis site of the left internal mammary artery (LIMA) to the left anterior descending coronary artery (LAD), in patients who had additional stenoses distal to the LIMA to LAD anastamosis site. They were able to demonstrate increased capillary density at the site of injection in patients who received FGF. In a three year follow up study, they were able to demonstrate an increased ejection fraction on echocardiography [80]. There were several other trials that emerged using both intra-coronary and intra-muscular routes of delivery of both VEGF and FGF either in protein form or through gene-delivery [81]. Unfortunately, in contrast to the Schumacher trial, which was a small trial with only 20 patients, several subsequent studies were negative, failing to show improvement on SPECT imaging and most importantly failing to demonstrate any improvement in exercise tolerance [8,82,83]. This is reflective of the short half-life of the protein and difficulty keeping a sustained concentration at the desired location.

In order to maintain a sustained protein release, researchers have attempted gene therapy. DNA plasmids were delivered often through viral vectors, especially adenovirus. Unfortunately several negative clinical trials highlight the challenges in achieving an efficient transduction rate [15,82,84,85]. Furthermore, unresolved issues of cytotoxicity, theoretical angioma formation, inflammation and potential risk of viral DNA incorporation into the host genome, continue to plague viral gene transfer, and limit its usefulness as the solution to neovascularization [86]. Non-viral vectors may avoid safety concerns, but may also encounter lower gene transfer efficiency, and lower gene expression time than their viral counterparts [87]. Currently, several therapeutic angiogenesis clinical trials are underway using gene therapy [17].

In addition, clinical trials have had modest success using cell-based therapy. In 2004, Wallert *et al.* demonstrated that intracoronary injection of autologous bone-marrow cells was safe in ST elevation myocardial infarction, and it produced a small but statistically significant increase in ejection fraction on cardiac MRI [88]. They were not able to uncover the mechanism, but suggested that it was likely secondary to paracrine effects including secretion of angiogenic factors in the peri-infarct zone.

With the incredible progress in neovascularization over the past decades, the next wave of clinical trials for therapeutic angiogenesis will incorporate more sophisticated protein and cell-based therapies with newer scaffold technology. However, attaining the goal of successful clinical trials requires tackling of the many challenges presented in this review; the more we learn about neovascularization, the more intricate and subtle the multistep process appears.

## 8. Conclusions

One of the great limiting factors to the advancement of tissue engineering and its clinical applicability is the ability to perfuse tissue engineering constructs. Therefore, enormous energy has been devoted to researching neovascularization. Over the last couple of decades, researchers have developed an increasing level of sophistication with manipulating pro-angiogenic factors and a plethora of delivery methods and scaffolds. The discovery of endothelial progenitor cells has opened the door to cell-based solutions to neovascularization. The future of this field will likely involve a combination of the strategies detailed in this article. This review article represents an overview of the literature presently available, and it is understood that development of neovasculature in engineered tissue will require much more research in regulating complex and dynamic interactions between angiogenic factors, stem cells, the factors secreted by cells, and the surrounding environment. Nevertheless, the studies presented here form a robust groundwork for further innovation.
